# The experiences and needs of unpaid family caregivers for persons living with dementia in rural settings: A qualitative systematic review

**DOI:** 10.1371/journal.pone.0286548

**Published:** 2023-06-14

**Authors:** Heather J. Campbell-Enns, Stephen Bornstein, Veronica M. M. Hutchings, Maya Janzen, Melanie Kampen, Kelli O’Brien, Kendra L. Rieger, Tara Stewart, Benjamin Rich Zendel, Malcolm B. Doupe

**Affiliations:** 1 Department of Psychology, Canadian Mennonite University, Winnipeg, Manitoba, Canada; 2 Department of Political Science, Memorial University, St. John’s, Newfoundland, Canada; 3 Faculty of Medicine, Memorial University, St. John’s, Newfoundland, Canada; 4 Aging Research Centre-NL, Grenfell Campus, Memorial University, Corner Brook, Newfoundland and Labrador, St. John’s, Canada; 5 School of Public Policy and Administration, Carleton University, Ottawa, Ontario, Canada; 6 Centre for Transnational Mennonite Studies, University of Winnipeg, Winnipeg, Manitoba, Canada; 7 St. Joseph’s Care Group, Thunder Bay, Ontario, Canada; 8 Western Health, Corner Brook, Newfoundland and Labrador, Canada; 9 School of Nursing, Trinity Western University, Langley, British Columbia, Canada; 10 Department of Community Health Sciences, University of Manitoba, Winnipeg, Manitoba, Canada; Tallinn University: Tallinna Ulikool, ESTONIA

## Abstract

**Purpose:**

Unpaid family caregivers provide extensive support for community-dwelling persons living with dementia, impacting family caregivers’ health and wellbeing. Further, unpaid family caregiving in rural settings has additional challenges because of lower access to services. This systematic review examines qualitative evidence to summarize the experiences and needs of rural unpaid family caregivers of persons living with dementia.

**Methods:**

CINAHL, SCOPUS, EMBASE, Web of Science, PsychINFO, ProQuest, and Medline were searched for articles investigating the experience and needs of rural family caregivers of persons living with dementia. Eligibility criteria were: 1) original qualitative research; 2) written in the English language; 3) focused on the perspectives of caregivers of community-dwelling persons with dementia; 4) focused on rural settings. Study findings were extracted from each article and a meta-aggregate process was used to synthesize the findings.

**Findings:**

Of the 510 articles screened, 36 studies were included in this review. Studies were of moderate to high quality and produced 245 findings that were analyzed to produce three synthesized findings: 1) the challenge of dementia care; 2) rural limitations; 3) rural opportunities.

**Conclusions:**

Rurality is perceived as a limitation for family caregivers in relation to the scope of services provided but can be perceived as a benefit when caregivers experience trustworthy and helpful social networks in rural settings. Implications for practice include establishing and empowering community groups to partner in the provision of care. Further research must be conducted to better understand the strengths and limitations of rurality on caregiving.

## Introduction

Dementia is an umbrella term for diseases characterized by the progressive deterioration of cognitive abilities and functional ability [1] that impact approximately 55 million people worldwide [2]. With 10 million new diagnoses every year, the global prevalence of dementia is projected to be 139 million people in 2050 [2]. While dementia is a key cause of dependency and disability for older adults [3], many persons with dementia live at home in community settings [4]. In Canada, for example, 61% of older persons with a dementia diagnosis live outside of institutional facilities (i.e., long-term care) [5]. While many of these individuals have mild or moderate dementias and low-care needs, some have complex care needs which can result in heavy caregiver burden. Canadian data show that 20% of community-dwelling persons with dementia have severe cognitive impairment, 25% exhibit responsive behaviours, 25% show signs of depression, and 28% require extensive assistance or are completely dependent on others for activities of daily living tasks like dressing and eating [5].

Unpaid family caregivers provide extensive support to community-dwelling people living with dementia. For example, they provide essential tasks such as supervision for persons with dementia and assistance with their activities of daily living. This care would account for over 40% of the total cost of dementia care if family caregivers were financially compensated for their work [6]. While most people living with dementia prefer to live at home, many are transferred to institutions when the care burden becomes too great for family caregivers [7].

While unpaid family caregivers recognize and report positive impacts of their caregiving [8], the demands of this care can negatively impact them by decreasing their overall psychological wellbeing and overall health [9], increasing health care use [9], and increasing chronic stress [4], social isolation and depression [10]. A recent systematic review [11] of family caregiver needs highlighted that they have specific needs (e.g., information, formal and informal support needs) related to the management of persons with dementia and also related to their own personal lives (e.g., helping to manage their own physical and psychological health) [11].

It is important to note that the geographical location where persons living with dementia and their caregivers reside influences their experiences and needs [12]. While definitions of rurality vary, most are based on population size or distance from services [12,13]. While rural unpaid family caregivers provide more hours of caregiving per week than their urban counterparts [14], it is unclear whether this is due to the inaccessibility or limited availability of formal services, the lack of other family and/or social support in rural settings, isolation exacerbated by geography and/or demographics, or some combination of these factors [12,15]. While family caregiver burden has been reported as being no different in rural areas than in urban areas, this may be because rural family caregivers have adapted to a lack of services by relying on informal supports [16]. Previous reviews on unpaid family caregiving in rural areas have not focussed on dementia care [13] or the qualitative experience of these caregivers [13,17].

New knowledge is needed to address gaps in dementia care literature, including the paucity of information on the experiences of family caregivers in rural communities, as well as their supportive care needs. Understanding how rurality influences family caregiving is necessary so family caregivers can be better supported, and family members can, in turn, better support persons living with dementia to live in their community settings as long as possible.

Accordingly, a systematic review was undertaken to address these gaps. A qualitative review was required to synthesize data across previous studies which represent the experiences of family caregivers in their own words. Thus, we conducted a systematic review of qualitative studies to summarize the experiences and needs of unpaid family caregivers living in rural settings while caring for persons living with dementia. Our overall question was “What are the experiences and needs of unpaid family caregivers of persons living with dementia in rural settings”? This review is intended to increase understanding of the family caregiver experience for health system leaders and health care providers who distribute resources to rural areas, as well as for organizations providing dementia education to families and communities. We also aim for this review to encourage further research to contribute to an expansive understanding of the experiences and needs of family caregivers in rural settings.

## Methods

A review protocol was registered and published with the PROSPERO database (ID: CRD42020163912). The PICo (population, phenomenon of interest, context) framework [18] was used to guide the development of this qualitative review. Accordingly, we focused on to the population of interest (family caregivers for persons living with dementia), the phenomenon of interest (experience and needs in relation to caregiver burden and stress and interactions with informal and formal care systems), and the context (rural setting). The Joanna Briggs Institute (JBI) method for qualitative systematic reviews was followed and a meta-aggregation method was used to collect and analyze the data [19]. Meta-aggregation is rooted in pragmatism and aims to examine qualitative evidence (e.g., experiences and meanings) and produce results in the form of generalizable statements applicable to policy or practice [19]. According to Lockwood et al. (2015), meta-aggregative reviews feature a defined objective or question, detailed inclusion and exclusion criteria, a comprehensive search strategy, quality appraisal of the included studies, analysis of data extracted, presentation and synthesis of findings, and transparency in the approach taken.

All authors participated in designing this review. The search strategy was designed in consultation with a university librarian. Three authors conducted the literature search, article selection, quality appraisal, data extraction, and synthesis of the data. An initial review took place from December 2019 to September 2020, but writing the review was paused due to the slowing of research activities during the COVID-19 pandemic. As a result, a second search was conducted in Feb 2022 to capture and add new literature. All authors provided feedback on the synthesis and were involved in writing the resulting report.

### Eligibility criteria

To be included, studies must have been original qualitative research written in English and focused on the experiences and needs of unpaid family caregivers of persons living with dementia in rural communities. Mixed-methods studies with a qualitative component were accepted if the qualitative data could be extracted for this review. Several terms were broadly defined to capture as many pertinent studies as possible: 1) “family” was defined as anyone a person living with dementia would consider to be family; 2) “caregiver” was defined as an unpaid family carer; 3) “dementia” captured both Alzheimer’s Disease and any other type of dementia; and, 4) “rural” included studies using the terms rural or remote to describe their settings. As well, studies were included with representatives of both rural and urban caregivers only in cases where the rural caregiver perspective could be extracted from the article.

Studies were excluded from this review if they did not: 1) focus on the experiences or needs of unpaid family caregivers of community-dwelling persons living with dementia; 2) include quotations from caregivers to support their findings; or, 3) focus on rural settings. Articles were also excluded if: 4) the full paper could not be found; and 5) the paper was not original research.

### Search

The following search strategy was developed in consultation with a university librarian, and the following terms were used consistently across the online databases: (dementia OR alzheimer* OR aging) AND (rural OR remote) AND (care* OR caregiver burden) AND (interview OR qualitative). Online databases were searched from their inception to December 2021, including CINAHL, SCOPUS, EMBASE, Web of Science, PsychINFO, ProQuest Dissertations & Theses, and MEDLINE. Google Scholar and Open Access Theses and Dissertations and other relevant websites were searched, as well as the reference lists of relevant articles and reviews.

### Article selection

All titles and abstracts were screened by two independent reviewers to determine if they met the inclusion criteria. Full text versions of potentially relevant studies were retrieved and independently assessed by two reviewers to confirm their eligibility. Disagreements were resolved through discussion and, in the case of disagreement or uncertainty on article inclusion, a third reviewer was consulted. All eligible articles were assessed using the JBI Critical Appraisal Tool for Qualitative Research.

### Data extraction

Authors developed a data extraction form to extract study details. The form was tested on three initial study articles and then revised as needed. Two authors extracted data independently and consolidated the data through discussion. If there was uncertainty, a third reviewer was involved. The form included study location, year of publication, purpose as stated by authors, methods, sample size, participant characteristics, setting, specific study findings, and an illustrative participant quotation for each study finding. The findings from each study were located and documented by examining the results sections of the included studies and were not derived through re-interpretation by this review.

### Data analysis and synthesis

Data analysis software (ATLAS.ti) was utilized to support the comparison of the extracted findings (e.g., themes) in the meta-aggregative process. All extracted findings (e.g., themes) and representative quotations were coded independently for their meanings and compared by three authors. Extracted findings were also coded for whether they explicitly referred to experiences or needs related to rural settings. Extracted findings were assembled into categories based on similarity of concept meaning (multiple findings per category) and the categories were then synthesized (with multiple categories being represented in each synthesized finding). These synthesized findings are overall descriptions of a group of categories derived from combining findings and aim to be representative of the evidence being combined [19].

## Results

### Study characteristics

**Fig 1:**
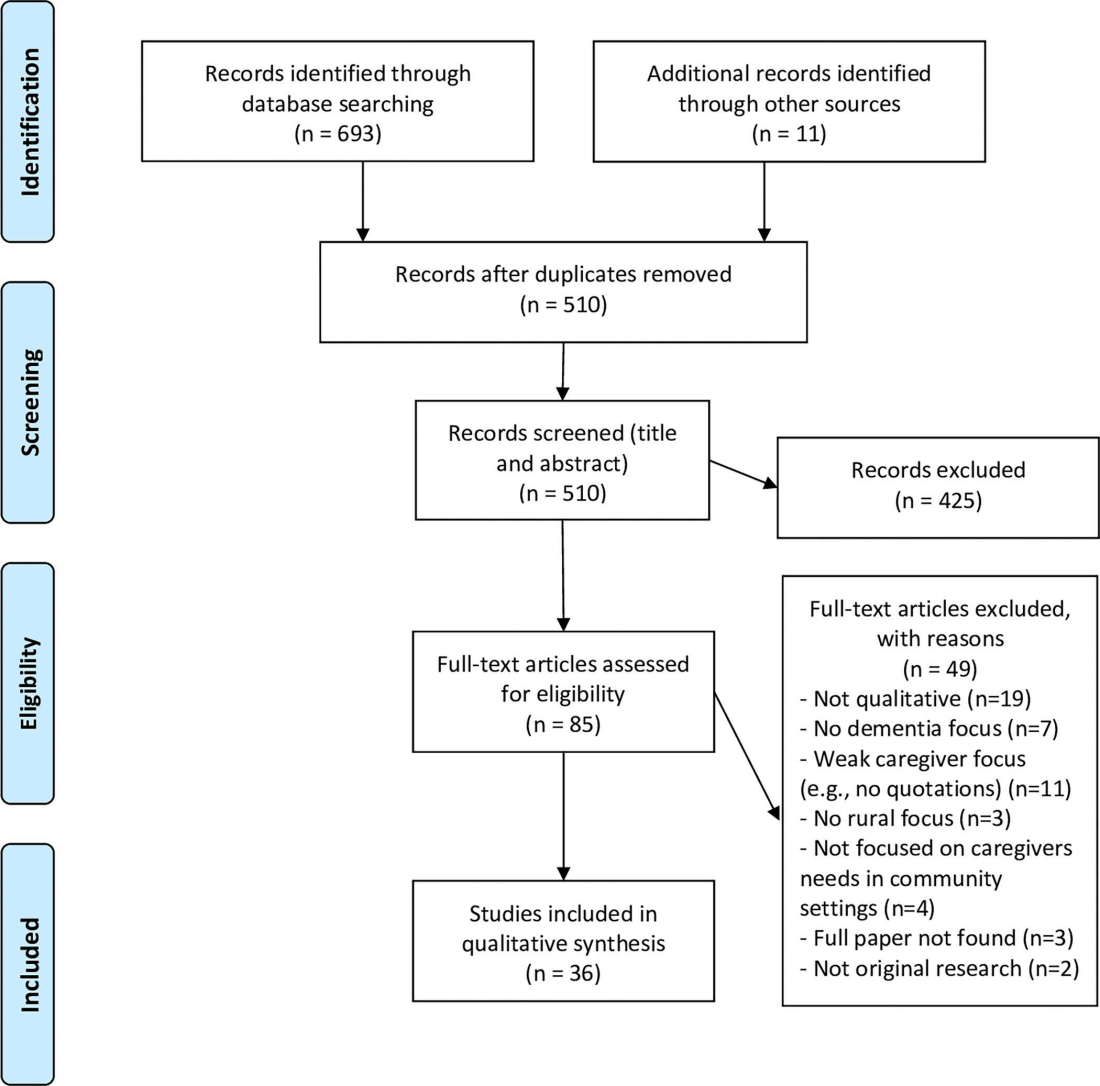
Flow diagram of research process.

The search strategy (Fig 1) retrieved 704 citations. After duplicates were removed, 510 articles were identified as potentially relevant to the objectives of the review. Titles and abstracts of the articles were examined, and 425 articles were excluded from the review because they did not meet the inclusion criteria. The full text of the remaining 85 articles were reviewed. After a full review, 49 further articles were excluded, and the remaining 36 articles were included in this review (Table 1) [20–53]. These 36 studies represented a total of 847 unpaid family caregiver participants (ranging from 3 to 166 participants). Thirty-four studies focused solely on rural caregivers and two studies included both rural and urban caregivers [32,38]. As reported in Table 1, studies were conducted by authors who were affiliated with institutions in Canada (n = 15), the United States (n = 7), Australia (n = 3), Scotland (n = 3), Norway (n = 1), Sweden (n = 1), South Africa (n = 1), Kenya (n = 1), Ghana (n = 1), and Japan (n = 1).

**Table 1:** Included studies and numbered findings.

Author(s), year, country	Purpose	Methodology; methods	Family caregivers	Findings	Quality rating
Agyeman et al. [20], 2019, Ghana	To explore the sociocultural beliefs, understandings, perceptions, and behaviours relating to living with dementia.	Qualitative; semi-structured focus groups; inductive thematic analysis	n = 18; gender/sex data are unclear; age data are unclear	1. Symptoms 2. Understanding of cognitive symptoms 3. Help seeking 4. Course of cognitive illness 5. Care arrangements 6.Economic impacts of care 7. Stigma	7/10
Arai et al. [21], 2000, Japan	To determine whether opinions of others may discourage the use of public services for the elderly.	Mixed methods; semi-structured interviews and thematic analysis	n = 7; gender/sex data are unclear; age data are unclear	8. Fear of not being deemed a dutiful daughter-in-law 9. Stigma attached to public services 10. Invasion of privacy	6/10
Blackstock et al. [22], 2006, Scotland	To develop a qualitative understanding of people with dementia and their carers’ experiences of using services.	Qualitative; semi-structured interviews, focus groups, field notes and thematic analysis	n = 30; 22 women, 8 men; age range: 40–90 years	11. Local social networks as informal support services 12. Supportive communities: Alternative perspectives 13. Personal relationship to place and space 14. Qualifying rurality: Implications of time and space	7/10
Branger et al. [23], 2016, Canada	To describe how rural caregivers cope with caring for a loved one diagnosed with mild cognitive impairment or dementia.	Qualitative description; an open-ended question on a survey and qualitative content analysis	n = 166; gender/sex data are unclear; age data are unclear	15. Social support: Help with planning and decision-making 16. Social support: Professional services 17. Time for self: Relaxing activity 18. Time for self: Physical activity 19. Time for self: Work and career 20. Time for self: Routine 21. Restructuring: Behavioural changes 22. Restructuring: Cognitive changes 23. Faith and religion 24. Joint or reciprocal activity 25. Checking in	7/10
Cox et al. [24], 2019, Australia	To illustrate (Australian) Aboriginal community understandings of dementia and their responses to dementia care.	Community participatory action research (two-stage mixed methods approach); individual interviews and thematic analysis	n = 12; gender/sex data are unclear; age data are unclear	26. Community understanding of dementia 27. Dementia care as cultural obligation 28. Dementia care support	8/10
Di Gregorio et al. [25], 2015, Canada	To explore the stages of the dementia journey through the viewpoints of health services providers, caregivers, community members, and people living with dementia.	Qualitative; individual interviews, group interviews and thematic analysis	n = 15; gender/sex data are unclear; age data are unclear	29. Community awareness of dementia 30. Receiving a diagnosis 31. Progression to long term care	8/10
Duggleby et al. [26], 2009, Canada	To explore the experience of home for family members caring for a person with dementia.	Grounded theory; Open-ended interviews and constructivist grounded theory approach to analysis	n = 17; 14 females, 3 males; age range: 48–84 years	32. Social context: The everyday experience 33. Fading hope as the main concern 34. Renewing everyday hope 35. Coming to terms 36. Finding positives 37. Seeing possibilities	8/10
Ehrlich et al. [27], 2017, Sweden	To explore and better understand the interrelationship between the caregiving experience of family caregivers and the sociocultural sphere that urban and rural areas represent.	Hermeneutical approach; open-ended interviews and hermeneutical analysis	n = 23; 9 females, 3 males; age range: 48–83 years	38. Accepting the course of life 39. Preserving normalcy 40. Fulfilling obligations	9/10
Forbes & Blake et al. [28], 2013, Canada	To enhance understanding of the process of knowledge sharing among health care practitioners, caregivers, and persons with dementia within a rural First Nations community.	Constructivist grounded theory; interviews, field notes, memos, and grounded theory analysis	n = 3; gender/sex data are unclear; age data are unclear	41. Developing trusting relationships: Supporting the person with dementia 42. Developing trusting relationships: Setting the stage 43. Developing trusting relationships: Resolving conflicts 44. Accessing and adapting the information: Accessing information during the early stages of dementia 45. Accessing and adapting the information: Accessing information during the late stage of dementia 46. Accessing and adapting the information: Accessing information from a variety of sources 47. Accessing and adapting the information: Inequitable resources in the community 48. Applying the information: Applying health promotion strategies 49. Involving caregivers in the decision-making	8/10
Forbes & Finkelstein et al. [29], 2012, Canada	To examine information needs, how these change over time, and how they access, assess, and apply the knowledge.	Qualitative interpretive description; interviews and thematic analysis	n = 14; 11 females, 3 males; age range: 39–91 years	50. Recognizing the symptoms 51. Receiving a diagnosis 52. Loss of independence 53. Initiating and using respite programs 54. Long term care facility placement 55. End of life care 56. Barriers to knowledge exchange: Lack of adequate rural community-based services and supports 57. Facilitators of knowledge exchange: Collaborating with team members 58. Expertise: Trusting relationships	8/10
Forbes & Markle-Reid et al. [30], 2008, Canada	To explore the use and satisfaction with home and community-based service for persons with dementia from the perspectives of caregivers.	Qualitative; focus groups, semi-structured interviews, and thematic analysis	n = 39; 82% females, 18% males; age data are unclear	59. Availability of home and community-based services 60. Acceptability of home and community-based services: Comprehensive assessments, treatment, and provision of dementia care 61. Acceptability of home and community-based services: Inconsistency of care provider 62. Acceptability of home and community-based services: Attributes of trusting partnerships 63. Acceptability of home and community-based services: Inflexible care 64. Acceptability of home and community-based services: Cost of services	8/10
Gibson et al. [31], 2019, United States	To conduct an inductive, thematic, analysis focusing on rural caregivers’ perceptions of familial and community supports.	Qualitative; semi-structured interviews and thematic analysis	n = 11; 81.8% females, 18.2% males; age range: 57–84 years	65. Rural caregivers on caregiving role, expectations, and values Helpful resources 66. and supports for rural caregivers 67. Unmet needs and unhelpful resources for rural caregivers	7/10
Gray-Vickrey [32], 1993, United States	To describe the lived experience of caring for a spouse with Alzheimer’s Disease.	Phenomenology; focus groups, semi-structured interviews, and phenomenological analysis	n = 11; 8 females, 3 males; age range: 41–87 years	68. Caretaking: Obligations and rewards of providing care 69. Caretaking: Caregivers are faced with problem behaviours 70. Caretaking; Emotions regarding caregiving 71. Caretaking: Prisoners in their own homes 72. Caretaking: No time or space to themselves 73. Caretaking: Financial burden 74. Caretaking: Caregivers feel constantly tired 75. Caretaking: Role change 76. Caretaking: Physical and emotional consequences of caregiving 77. Caretaking: Caregiving is time consuming 78. Caretaking: Coping with problem behaviours 79. Caretaking: Distancing 80. Marital relationship: Loss of companionship 81. Marital relationship: Caregiver’s distress at watching spouse’s decline 82. Changes in social support: Others fail to recognize the caregiver’s burden 83. Changes in social support: Caregiver is the other victim 84. Changes in social support: Social isolation 85. Changes in social support: Lack of appropriate or adequate informal support 86. Changes in social support: Caregiver perceptions of satisfactory informal support 87. Changes in social support: Support from support groups 88. Changes in social support: Conflicting feelings about the health care delivery system 89. Changes in social support: Inadequacy of nursing homes 90. Changes in social support: Home and health aide support 91. Understanding the Alzheimer’s process: Awareness of the disease process 92. Understanding the Alzheimer’s process: Knowledge about Alzheimer’s Disease 93. Understanding the Alzheimer’s process: Concern about the future	8/10
Gurayah [33], 2012, South Africa	An exploratory study into the phenomenon of caregiving for people with dementia in a rural context in South Africa.	Phenomenology; semi-structured interviews and thematic analysis	n = 5; 4 females, 1 male; age range: 46–68 years	94. Views and responsibilities of the caregiver 95. Impact of caregiving 96. Services to assist the caregiver	8/10
Herron & Rosenberg [34], 2017, Canada	Examine how people with dementia experience their communities and support within them.	Qualitative case study approach; semi-structured interviews, field notes and constant comparison approach to analysis	n = 43; gender/sex data are unclear; age data are unclear	97. Recognizing community connections and contributions 98. Negotiating space outside the home 99. Identifying care needs and negotiating service use	7/10
Herron & Rosenberg [35], 2019, Canada	Examine experiences of providing and accessing care over the course of dementia and across settings.	Qualitative case study approach; semi-structured interviews and constant comparison approach to analysis	n = 27; gender/sex data are unclear; age range: 46–89 years	100. Navigating the system 101. Finding people who understand 102. Seeking home care hours 103. Resistance to respite 104. Making decisions about end-of-life care	7/10
Herron, Rosenberg & Skinner [36], 2016, Canada	To explore how partners in care negotiate support over time and across different settings and the major challenges over the course of the condition.	Mixed methods; in-depth structured interviews and constant comparison approach to analysis	n = 43; gender/sex data are unclear; age data are unclear	105. Rural experiences of voluntary sector service	7/10
Herron & Skinner [37], 2013, Canada	To make visible the over-looked emotional experiences of older rural people and their carers, and to reveal the unjust conditions of care within the rural context.	Qualitative; in-depth interviews, focus groups and thematic analysis	n = 14; 10 females, 4 males; age range: 50–80 years	106. Interpersonal challenges 107. Caring places: Feeling at home	7/10
Hughes et al. [38], 2009, United States	To examine the experiences of African American caregivers that led them to seek a formal diagnosis for a family member.	Qualitative; semi-structured interviews and grounded theory analysis	n = 17; 14 females, 3 males; age range: 42–80 years	108. Participants’ prior knowledge of Alzheimer’s disease 109. Perceptions of perceived severity of Alzheimer’s disease 110. Perceptions of susceptibility to Alzheimer’s disease 111. Perceptions of facilitators and barriers to a diagnosis 112. Perceptions of benefits to a diagnosis 113. Cues to action 114. Perceptions of self-efficacy	7/10
Innes et al. [39], 2005, Scotland	To develop a qualitative understanding of service use from the point of view of people with dementia and their carers in rural Scotland.	Qualitative; semi-structured interviews, focus groups and use of a coding structure.	n = 30; 22 females, 8 males; age data are unclear	115. Gaps in services available: Transport 116. Gaps in services available: Respite provision 117. Gaps in services available: Support for informal carers 118. Gaps in services available: Home care 119. Gaps in services available: Day care 120. Positive experiences of service provision: Services appropriate for needs 121. Positive experiences of service provision: Loving care 122. Positive experiences of service provision: Social life 123. Positive experiences of service provision: Stimulating 124. Positive experiences of service provision: Relationship with service providers 125. Positive experiences of service provision: Support 126. Positive experiences of service provision: Free time 127. Positive experiences of service provision: peace of mind 128. Positive experiences of service provision: Trained staff 129. Positive experiences of service provision: Service provider relationship with carer	7/10
Innes et al. [40], 2014, Scotland	To explore the reported difficulties and satisfactions and diagnostic processes and post-diagnostic support offered to people with dementia and their families living in the largest remote and rural region in Scotland.	Qualitative; semi-structured interviews and thematic analysis	n = 12; 11 females, 1 male; age range: 45–80 years	130. Pre-diagnosis: Recognising the problem and seeking help 131. Pre-diagnosis: Rationalization and denial 132. Experience of the diagnostic process 133. Experience of the diagnostic process: Delivery of the diagnosis 134. Experience of the diagnostic process: Reaction to the diagnosis 135. Post-diagnostic support: The needs of service users 136. Post-diagnostic support: Satisfaction with services 137. Post-diagnostic support: Support for carers 138. Post-diagnostic support: The need for information	7/10
Larsen et al. [41], 2017, Norway	To explore how formal and family caregivers experience collaboration while providing home-based dementia care, with a focus on user participation.	Qualitative; semi-structured interviews and thematic analysis	n = 7; gender/sex data are unclear; age data are unclear	139. Negotiating participation in decisions: They no longer know what is best for them 140. Negotiating participation in decisions: The person living at home is the boss 141. Negotiating participation in decisions: Acute necessary health care 142. Negotiating formal care intervention: Family caregivers want to preserve normality Negotiating formal 143. care intervention: Family caregivers’ care burden breakdown 144. Negotiating the right to speak on behalf: Family caregivers fight for resources 145. Negotiating the right to speak on behalf: The most troubling issue	8/10
Lilly et al. [42], 2012, Canada	To investigate the health and wellness and support needs of family caregivers to persons with dementia in the Canadian policy environment.	Qualitative; focus group and thematic analysis	n = 19; 16 females, 3 males; age range unclear	146. Forgotten: Abandoned to care, alone and indefinitely 147. Information and referral 148. Adequate and appropriate in-home service provision for their care recipient 149. Respite and relief services for caregivers 150. Assistance with care recipients’ transitions into long term care 151. Unrealistic expectations for caregiver self-care	7/10
Mattos et al. [43], 2019, United States	To explore perceived social determinants of health among older, rural-dwelling adults with early-stage cognitive impairment.	Qualitative description; semi-structured interviews and thematic analysis	n = 10; 7 females, 3 males; age range: 64–78 years	152. Staying active 153. Eating well 154. Living with cognitive changes 155. Advantages of living rural 156. Disadvantages of living rural 157. Relying on children 158. Connecting with neighbours and community	8/10
Morgan et al. [44], 2002, Canada	To describe the community-based process and pilot project used to develop a study of rural dementia care.	Mixed methods; focus group and thematic analysis	n = 4; 4 females; age data are unclear	159. Stigma of dementia 160. Acceptability and accessibility of services 161. Service delivery challenges 162. Consequences of not using support services	7/10
Musyimi et al. [54], 2021, Kenya	To explore perceptions and experiences of dementia and related care in rural Kenya	Qualitative; in-depth interviews and focus groups; inductive thematic analysis	n = 12; 8 females, 4 males; age range: 25–86 years	163. Negative stereotypes of dementia: Negative labels attached to dementia 164. Negative stereotypes of dementia: Traditions and cultural beliefs 165. Negative stereotypes of dementia: Normal ageing and ageism 166. Limited knowledge about dementia care and treatment: Carers’ knowledge 167. Diagnostic pathway 168.Neglect and abuse	7/10
Nguyen et al. [55], 2021, Vietnam	To describe the meanings of dementia and the day-to-day lived experience of family caregiving in an area just outside of central Hanoi.	Descriptive qualitative; interviews; thematic analysis	n = 12; 6 females, 6 males; age range: 48–62 years	169. Perceptions of dementia 170. Family caregiving as moral obligation 171. Gender and birth order in family caregiving 172. Difficulties and challenges of family caregiving: Time demands 173. Difficulties and challenges of family caregiving: Loss of income 174. Difficulties and challenges of family caregiving: Worsening of physical health 175. Difficulties and challenges of family caregiving: Increased social isolation 176. Difficulties and challenges of family caregiving: Emotional distress	8/10
Orpin et al. [45], 2014, Australia	To explore patterns of formal and informal support utilisation by people caring for a person with dementia in a rural-regional context.	Unstated methodology; semi-structured interviews and thematic analysis	n = 18; 8 females, 10 males; age range: 77–83 years	177. The rural context 178. The primary carer role 179. Patterns of carer support	3/10
Prorok et al. [46], 2017, Canada	To examine the perceived primary care health care experiences of both PWD and their caregivers in Ontario, Canada, using qualitative methods.	Qualitative; focus groups and thematic analysis	n = 21; 14 females, 7 males; age range: 45–81 years	180. Communication: Content communicated 181. Communication: Day to day management 182. Communication: Long term management 183. Communication: Managing self 184. System navigation: ‘Point person’ necessary 185. System navigation: Prolonged path to resources and supports 186. Ease of access: Timing 187. Ease of access: Provider knowledge	7/10
Sanders [47], 2007, United States	To examine the experience of male caregivers with their informal support networks.	Phenomenology; semi-structured interviews and phenomenological analysis	n = 20; 20 males; age range: 41–84 years	188. Perception of the willingness of the informal support networks to provide help: Not involved with care 189. Perception of the willingness of the informal support networks to provide help: Emergency assistance only 190. Perception of the willingness of the informal support networks to provide help: Feel free to call if we could be of help 191. Perception of the willingness of the informal support networks to provide help: Part of the caregiving team 192. Willingness of the male caregiver to ask for help: Asked for assistance 193. Willingness of the male caregiver to ask for help: Felt guilty about asking for help 194. Willingness of the male caregiver to ask for help: Did not ask for help	8/10
Sanders & McFarland et al. [48], 2002, United States	To determine what factors lead sons to assume a primary caregiver role for a parent with progressive memory loss and the caregiving challenges most commonly experienced.	Grounded theory; unstructured interviews and use of coding processes	n = 18; 18 males; age range: 35–67 years	195. Initial reaction to memory loss 196. Becoming the caregiver 197. Women in the sons’ world 198. Learning new roles 199. Conflicts 200. Uncomfortable situations 201. Service utilization	8/10
Sanders & Power et al. [49], 2009, United States	To examine the changes that occur in the roles, responsibilities, and relationships husbands who provide care for their wives with memory loss and other chronic health conditions.	Phenomenology; semi-structured interviews and phenomenological analysis	n = 17; 17 males; age range: 66–85 years	202. Adaptation of old roles to new roles due to increased responsibility: Protector of self-esteem, dignity, and personhood 203. Adaptation of old roles to new roles due to increased responsibility: Provider of personal care 204. Adaptation of old roles to new roles due to increased responsibility: Planner of activities and socialization 205. Adaptation of old roles to new roles due to increased responsibility: Home maintenance and keeper 206. Developing new relationships with their wives: Developing a new type of intimacy and closeness 207. Developing new relationships with their wives: Adjusting to the personality changes associated with chronic illness 208. Developing new relationships with their wives: Learning to cope with the unexpected in their relationship 209. Developing new relationships with their wives: Recognizing the finality of the relationship	8/10
Smith, A. et al. [50], 2001, United States	To learn more about the process involved in living as a primary caregiver of an Alzheimer’s patient. In addition, the goal was to learn more about the needs of caregivers in the struggles they faced.	Qualitative; semi-structured interviews and analysis methods were unclear	n = 45; 39 females, 6 males; age data are unclear	210. Financial assistance 211. Legal assistance 212. Medical assistance 213. Housing assistance 214. Emotional support/assistance 215. Spirituality	5/10
Smith, K. et al. [51], 2011, Australia	To determine ways to overcome factors affecting the successful delivery of services to Aboriginal people with dementia living in remote communities, and to their families and communities.	Qualitative; in-depth interviews and thematic analysis	n = 31; gender/sex data are unclear; age data are unclear	216. Caregiver role: Reasons for being a caregiver 217. Caregiver role: additional care giving responsibilities 218. Caregiver role: Sharing care-giving role 219. Perspectives of dementia: Causes of dementia 220. Perspectives of dementia: Signs and symptoms 221. Community and culturally appropriate care: Community engagement 222. Community and culturally appropriate care: Community-based care 223. Community and culturally appropriate care: Culturally appropriate activities 224. Workforce: Aboriginal staff 225. Workforce: Staff that are trusted and accepted 226. Workforce: Local support and guidance 227. Education and training: Dementia training 228. Issues affecting remote communities: Transport 229. Service issues: 229. Communication and coordination 230.Services issues: Caregiver support services	7/10
Vellone et al. [52], 2012, Canada	To explore the meaning of quality of life for Sardinian caregivers of people affected with Alzheimer’s disease and factors improving and worsening their quality of life.	Phenomenology; open-ended interviews and extraction of themes	n = 41; 35 females, 6 males; age range: 26–78 years	231. What is quality of life? Unity and cooperation in the family 232. What is quality of life: Freedom, independence, having time for themselves 233. What is quality of life? Serenity/tranquility 234. What is quality of life? Well-being and health 235. Factors worsening quality of life–fear for the future: For the care needed and for the illness and worsening 236. Factors worsening quality of life–continuous care of the patients: not having time for themselves 237. Factors improving quality of life: No worsening of the illness 238. Factors improving quality of life: Help and support from family 239. Factors improving quality of life: Help from formal services 240. Factors improving quality of life: Satisfaction and reward from giving care 241. Factors improving quality of life: Financial support for paying for assistance 242. Factors improving quality of life: More free time 243. Factors improving quality of life: More public sensitization about Alzheimer’s disease	9/10
Wiersma & Denton [53], 2016, Canada	To explore and understand the context of dementia in rural northern communities in Ontario with an emphasis on understanding how dementia friendly the communities were.	Qualitative interpretivist constructionist paradigm; in-depth interviews, field notes and coding processes for analysis	n = 15; gender/sex data are unclear; age data are unclear	244. Looking out for the person with dementia 245. Remaining connected	8/10

### Study quality

The quality appraisal checklist consisted of 10 items, and, except for one low-quality article, all the studies were of moderate to good quality overall. No article included all 10 items (average = 7.1 of 10; range = 3 to 9 of 10) (Table 1). The most frequently excluded quality items were a statement locating the researcher culturally or theoretically (n = 35), the influence of the researcher on the research and vice-versa (n = 32), and a stated philosophical perspective and research methodology (n = 21).

We also report on whether and how rurality was defined. In half of the studies, the authors did not objectively define this term [21,24,26,28,30,31,33,38,41,46–52,54,55]. In the remaining studies, authors defined rurality by various criteria–geographic location [36,39,40], proximity to urban centres or distance to services [23,29,34,37,43,45], population density [27,42], by the agricultural or resource-based economy of the area [20,35], or the perceived rural identity of participants [34,36]. As well, several studies used a combination of the above factors in defining rurality; various studies utilized a combination of population density and proximity to urban centres [22,25,32,53], a combination of population density and type of economy [44], or a combination of geographic location, economic factors, and perceived rural identity [36].

### Synthesized findings

Data extraction resulted in 245 study findings from the 36 included articles. Extracted findings were numbered to track their contributions to the synthesized findings (Table 1). After coding each extracted finding, codes were aggregated into 11 categories. These categories were 1) coping, 2) stress and emotions, 3) finances, 4) role/identity of caregiver, 5) noticing health changes, 6) family support, 7) getting help and/or adapting, 8) communication, 9) decision-making, 10) services provided and, 11) information and understanding. Then, through a process of reading and re-reading category contents, categories were then synthesized into three synthesized findings to describe the unpaid family caregiver experience, with two findings particular to the rural setting. Table 2 shows how categories contributed to synthesized findings by referring to extracted finding numbers. No extracted findings were omitted from the synthesis. The three synthesized findings are described below.

**Table 2:** Finding numbers related to each synthesized finding and category.

Synthesized finding	Category	Finding number (as in Table 1)
The challenge posed by dementia	Coping	17–24, 48, 65, 77, 79, 86, 87, 134, 149–153, 181, 208, 242
	Stress and emotions	17–19, 31–34, 40, 45, 50, 58, 62, 63, 65, 68–72, 74–76, 78–81, 83, 84, 89, 90, 93, 95, 106, 110, 114, 117, 119, 130, 134, 140, 141, 143–146, 148–151, 162, 172, 174–176, 178, 179, 183, 199, 200, 204, 206–214, 217, 233–240, 242
	Finances	5, 6, 52, 53, 73, 75, 117, 172, 173, 182, 210, 211, 241
	Role/identity	72, 75, 94, 175, 198, 203, 205, 206, 208, 210, 232
	Noticing changes	21, 50, 60, 89, 91, 112, 113, 132, 133, 201, 207, 212, 213, 236
	Family support	5, 10, 25, 36, 39, 40, 43, 44, 46, 48, 49, 52, 55, 59, 82, 86, 114, 137, 154, 170, 171, 178, 186, 191–193, 195–198, 214, 216, 218, 226, 231, 238, 240
	Getting help/adapting	10, 21, 22, 35, 37, 38, 50, 57, 64, 94, 96, 97, 146, 147, 194, 201, 207, 213, 230, 236, 238
	Communication	42, 133, 138, 147, 167, 180, 183, 221
	Decision making	5, 43, 45, 49, 54, 55, 104, 139–141, 143
	Services provided	16, 41, 42, 44, 45, 47, 49–51, 54, 55, 57–63, 101, 103, 111, 112, 116, 120–126, 128, 131, 135, 137, 141, 143, 145–151, 161, 162, 178, 184, 185, 187, 191, 201, 212, 221, 225, 229, 239, 241
	Information/understanding	1–4, 16, 26, 29, 31, 35, 51, 91, 92, 108–112, 116, 132, 151, 163–166, 180, 184, 187, 191, 195, 201, 211, 212, 220–221, 227
Rural limitations	Stress and emotions	7, 8, 56, 88, 105, 159, 188, 202
	Role/identity	177
	Family support	8, 13–15, 28, 85, 98–100, 173, 188, 215
	Getting help/adapting	12, 13, 98, 99, 168, 188
	Services provided	9, 27, 28, 67, 88, 100, 102, 105, 107, 115, 118, 136, 159, 160, 169, 223, 224, 226, 228
	Information/understanding	7, 28, 30, 67, 88, 159, 160, 169, 202, 243
Rural opportunities	Coping	66
	Stress and emotions	127, 155, 156
	Family support	7, 11, 13, 66, 157, 158, 177, 222, 244, 245
	Getting help/adapting	11, 13, 66, 142, 158, 189, 190, 244, 245
	Communication	142
	Services provided	127, 128

### Synthesized finding 1: The challenge posed by dementia for rural caregivers

“The challenge posed by dementia” describes the difficult or demanding experiences related to being an unpaid family caregiver of a person with dementia, as perceived by participants living in rural areas. As participants did not explicitly link many of these experiences with providing unpaid care in rural settings, it is unknown if these experiences are related to, or exacerbated by, the rural context. Since this synthesized finding captures the overall experience of unpaid family caregiving, all 11 categories (Table 2) contributed to this synthesized finding.

First, unpaid family caregivers reported that seeking a dementia diagnosis was challenging. They met with healthcare providers who initially dismissed the possibility of a dementia diagnosis because the person with dementia was too young or because memory loss was an expected characteristic of older age [20,24,38,44,46,50,54]. Living with pre-diagnosed dementia was difficult for caregivers [25,29,38,40,54] and caregivers described the need for improved dementia information and education [20,23–26,29,32,38–40,42,46,48,50,51]. They also needed improved communication with and among healthcare providers to be better informed about dementia and the future course of the illness [28,40,42,46,50].

Second, unpaid family caregivers found formal healthcare services (e.g., home care) to be lacking, inflexible and/or uncoordinated [23,29,30,35,39–42,44–46,48,50,51]. As a result, caregivers were left to fill gaps in services by providing services themselves and/or by developing personal networks to help provide care [22,28,34–36,42,44,47]. To alleviate this burden, caregivers wished to have someone who could guide the process of facilitating connections among and across services and providers [40,46,51], especially when providing care became too burdensome for the caregiver to continue safely [41,42,44,48–50].

Third, unpaid family caregivers experienced negative emotions (e.g., sadness, anger) as a part of their caregiving and this was a source of stress [23,25,27–30,32,33,37–41,41,42,44–46,48–52,55]. While many caregivers found ways to cope with, or adapt to, these negative emotions by seeking support or taking time for self-care activities, participants often reported challenges with finding the support to alleviate their responsibilities and enable them to practice self care [23,28,30,32,42,43,46,49,52,55].

### Synthesized finding 2: Rural limitations

“Rural limitations” describes the barriers or limitations experienced by unpaid family caregivers for persons living with dementia when providing care in rural settings. Six categories contributed to this synthesized finding (stress and emotions, role/identity of caregiver, family support, getting help and/or adapting, services provided, information and understanding).

First, several studies reported that caregivers of persons living with dementia in rural communities felt stigmatized in their community and that the challenges they experienced were not understood by community members [20,22,25,44,45,52,54]. A lack of community member education about dementia (e.g., defining dementia, understanding dementia symptoms, understanding the needs of caregivers) was a challenge which precipitated negative emotions for caregivers [24,25,29,45,54]. Where formal community-based programming for persons living with dementia and their caregivers existed (e.g., day programs, support groups), caregivers noted that they perceived the programming to be more suitable for women than for men (i.e., for both caregivers and persons living with dementia) [34,50].

Second, unpaid family caregivers reported that rural communities had changed due to population aging and the departure of family members for urban areas. As rural communities changed over time, caregivers hesitated to ask for support from remaining community members who had their own health concerns and limitations, as fewer family members were available to provide them with support in rural settings [32,35,43,47,55]. Some caregivers felt that governments did not have the will to provide better supports to help older people age in place in rural areas [37].

Third, unpaid family caregivers felt that the quality of health services in rural communities was inferior to that provided in urban communities. Distance to specialized services led to transportation challenges and excessive time away from home to travel long distances [32,39,40,51]. Where services were provided in their rural communities, caregivers noted a lack of specialized knowledge about dementia (e.g., by family physicians, respite workers, and home care workers who lacked dementia training) [31,48,55] or that, even when a provider had some dementia training, they may not have knowledge appropriate to the specific community and/or culture. For example, participants noted a lack of Indigenous workers in communities with large Indigenous populations [24,51]. As well, rural communities experienced a lack of and/or high turnover in home care and respite workers resulting in inflexible care schedules, inconsistent care providers, and a lack of respite for caregivers [29,30,39,42,44,51,55].

### Synthesized finding 3: Rural opportunities

Study participants often described the benefits and opportunities available for persons living with dementia in rural communities and their unpaid family caregivers. Seven categories contributed to this synthesized finding (coping, stress and emotions, family support, getting help and/or adapting, communication, services provided, information and understanding).

Participants most often cited the strong personal networks in close-knit rural communities and how these affected them positively when caregiving for someone with dementia. These networks are not capable of providing specialized dementia services, but they involve trusting relationships and feelings of mutual responsibility based on the specific local environment. At times, rural environments were described as positive because of the beauty of the landscape [22], yet positive comments were more often associated with caregivers’ feelings of trusting others, being known by others in their close-knit communities, and having community members help supervise persons living with dementia in the community [22,27,31,45,53].

Even where dementia-specific care might not be ideal because of distance from services and local challenges [22], caregivers described the rural setting as a place where they have ties to others, even when rural populations are shrinking [22,45,53]. As a result, participants felt that rural communities provided them with a sense of safety and opportunity when caring for persons living with dementia. A rural network provided a safety net when there was a personal connection to someone who had healthcare knowledge (e.g., a retired nurse), leading caregivers to feel they had someone whom they could call on in a crisis [47].

Family caregivers also felt that rural settings provided opportunities for persons living with dementia. Persons living with dementia were able to link with people and places to participate in activities that provided continuity with their lives before diagnoses (e.g., repairing mechanical parts, gardening, being outdoors with others) [28,31,34]. This was reported as enjoyable and meaningful for persons living with dementia and was beneficial for caregivers because it provided a form of respite for caregivers.

## Discussion

Given the growing incidence of dementia in rural and urban settings, it is important to understand how rurality affects the experiences and needs of caregivers of persons living with dementia in rural communities u. This systematic review of qualitative research uniquely summarizes the experiences and needs of rural family caregivers of persons living with dementia. A meta-aggregate approach was used to establish the synthesized results via coding and categorizing each finding from all included studies and then synthesizing the categories.

These synthesized findings provide new knowledge about unpaid family caregivers’ perspectives relating to the limitations and opportunities of living in rural areas while providing care for persons living with dementia. The findings reveal the challenges posed by dementia from the perspectives of caregivers as well as ways in which rural settings may pose limitations and/or provide opportunities when caring for community-dwelling persons living with dementia.

Of note is the importance of relationships to unpaid family caregivers in rural settings. On one hand, caregivers described this setting as limiting due to experiences of stigma yet, on the other hand, the rural setting also benefited caregivers when they experienced trustworthy and helpful relationships in close-knit communities. The importance of addressing stigma in rural settings is documented by others [56,57]. This present review both supports and enriches these findings by demonstrating that unpaid family caregivers may experience stigma in rural communities while, at the same time, often appreciate and rely on close-knit rural relationships. These important relationships are built on small social networks that can help to foster feelings of comfort and safety in rural areas. They also provide crucial opportunities for persons living with dementia to participate in meaningful activities while being supervised by others, thus providing a form of informal respite for unpaid family caregivers when formal respite services may not be available. This is significant in that trustworthy and helpful relationships in rural communities may be protective for unpaid family caregivers. While rural living contributes to challenges for caregivers, this review also captures the experience of rural living as an asset due to the existing social networks in these settings.

This review has implications for health systems and organizations. If unpaid family caregivers experience their communities to be potential assets in the provision of dementia care, health systems and health organizations could engage and empower existing social networks in rural communities to reduce dementia-related stigma and to co-produce dementia care with community partners. This co-production of care may be facilitated by integrated community care, an approach to care which values the role of the informal care sector, including family caregivers [58]. Intersectoral partners have a role in linking dementia education to communities, increasing access to technology in rural areas through better infrastructure, increasing funding for community-driven programs, and developing local and regional policies to increase community involvement in care.

Implications for research include the need to further investigate both the positive and the negative impacts of rural life on unpaid family caregiving, including identifying outcomes most important to caregivers in rural settings and examining factors that can improve outcomes. Investigating these outcomes and factors will facilitate the development of needed interventions in rural contexts. Methodologically, research using a community-based research approach [59] will build capacity in rural settings for unpaid family caregivers and their communities to direct research projects to best meet their needs and to involve local and regional governments in improving outcomes for caregivers in rural settings.

### Limitations

This review is limited in that the definition of rurality was inconsistent or lacking across studies reviewed, and we cannot know how differing conceptualizations of rurality may have impacted this review. Second, the included studies vary in quality which may have impacted our findings. Lastly, each finding is associated with the experiences and needs of caregivers of persons living with dementia in rural communities, yet we do not directly compare the experiences of rural and urban caregivers in this review. As such, we cannot claim these findings to be unique to rural caregivers, yet our meta-aggregate analysis demonstrates that rural caregivers attribute some of their unmet needs, as well as some of their supports, specifically to their rural contexts.

## Conclusion

This meta-aggregate analysis of qualitative studies describes the experience and needs of rural unpaid family caregivers for persons living with dementia. We have identified three synthesized findings. The first synthesized finding describes the challenges posed by dementia for caregivers, the second describes how caregiving is limited in rural settings, and the third describes the opportunities experienced by family caregivers due to their rural settings. We suggest healthcare systems build on the strengths of rural areas and work with communities to provide dementia care. We also call for future research to utilize community-based approaches to identify outcomes important to rural caregivers.

## Supporting information

S1 Checklist PRISMA 2009 checklist. (DOC)
