# Supplementary material for: The experiences and needs of unpaid family caregivers for persons living with dementia in rural settings: A qualitative systematic review
Source: PLoS One. 2023 Jun 14;18(6):e0286548. doi: 10.1371/journal.pone.0286548 (PMC10266592; doi:10.1371/journal.pone.0286548)
Supplement: Attachment — Submitted filename: Reviewer Suggestions (1).docx [file pone.0286548.s002.docx]

**Review Report:**

**Article Title:**

**The experiences and needs of unpaid family caregivers for persons living with dementia in rural settings: A qualitative systematic review**

Overall, it is a very well written manuscript, it is acceptable for publication however; a few suggestions are as follows:

- In paper, a cohort of studies was selected as there is only one study published in 1993 which is mentioned in Table (1), while the remaining 35 studies are from 2000-2022. How was this cohort decided? If researchers intended to add research published in 2000-22 then they should exclude 1993.
- In paper, study rationale and implication are missing. Add some paragraphs about study implications and significance.
- Researchers selected studies from Canada (n=15), the 187 United States (n=7), Australia (n=3), Scotland (n=3), Norway (n=1), Sweden (n=1), South Africa 188 (n=1), Kenya (n=1), Ghana (n=1), and Japan (n=1). As Japan is an Asian country then researches from other Asian countries like Pakistan, India, Sri Lanka, China, Iran, Malaysia, were not included? kindly write down the reasons behind selection of countries.
- Researchers just gave overall findings of researches, at least they should specify the category of caregiver, no doubt, in method section, they have described caregiver” was defined as an unpaid career who may be referred to as a care partner or informal career; But in Table 1, a separate column should be made for the extracted themes to elaborate that family caretaker might have different concerns than those informal careers.
- Selected studies did not specify that patients with dementia were suffering from other morbidities or not, and also did not explain that patients might be completely paralyzed or partially paralyzed or were quite stable if in the early stage of dementia.
- Studies did not specify the age of caregivers and gender. Age and gender also bring different responses.
- In discussion, there is a need to describe findings from formal (family members) and informal careers separately.
