# Supplementary material for: The experiences and needs of unpaid family caregivers for persons living with dementia in rural settings: A qualitative systematic review
Source: PLoS One. 2023 Jun 14;18(6):e0286548. doi: 10.1371/journal.pone.0286548 (PMC10266592; doi:10.1371/journal.pone.0286548)
Supplement: Attachment — Submitted filename: Response to Reviewers - PLOS ONE - 2023-05-04.docx [file pone.0286548.s003.docx]

Editors, PLOS ONE

RE: PONE-D-23-02151 Response to Reviewers

May 4, 2023

Dear Editors,

Thank you for the opportunity to respond to reviewer feedback for our manuscript entitled, *The experiences and needs of unpaid family caregivers for persons living with dementia in rural settings: A qualitative systematic review.*

Responses to the editor and reviews are included in the sections below.

A. **Responses to points raised by the editor:**
1. In paper, a cohort of studies was selected as there is only one study published in 1993 which is mentioned in Table (1), while the remaining 35 studies are from 2000-2022. How was this cohort decided? If researchers intended to add research published in 2000-22 then they should exclude 1993.

Response: There were no study exclusions based on year of publication. The 1993 study was a dissertation that resulted from our searches, and it was highly relevant to the purpose. All other studies were published from 2000 and on, but there was no reason to exclude the 1993 study based on the eligibility criteria.

2. In paper, study rationale and implication are missing. Add some paragraphs about study implications and significance.

Response: Thank you for the opportunity to provide more information. Please see lines 96-112 for rationale, implications, and significance.

3. Researchers selected studies from Canada (n=15), the 187 United States (n=7), Australia (n=3), Scotland (n=3), Norway (n=1), Sweden (n=1), South Africa 188 (n=1), Kenya (n=1), Ghana (n=1), and Japan (n=1). As Japan is an Asian country then researches from other Asian countries like Pakistan, India, Sri Lanka, China, Iran, Malaysia, were not included? kindly write down the reasons behind selection of countries.

Response: No countries were excluded from this systematic review. There were no other countries represented in this list because there were no more studies which met the inclusion criteria.

4. Researchers just gave overall findings of researches, at least they should specify the category of caregiver, no doubt, in method section, they have described caregiver” was defined as an unpaid career who may be referred to as a care partner or informal career; But in Table 1, a separate column should be made for the extracted themes to elaborate that family caretaker might have different concerns than those informal careers.

Response: This systematic review is solely on the experience of family caregivers, also described as unpaid family caregivers or carers. A change was made to line 142 to be clearer. (We recognize the confusion that results when there is no one term for unpaid family caregivers and the term “informal” caregiver can mean a paid person outside the family. Thus, the article is only addressing family members and the reference to care partner or informal caregiver was removed for ease of understanding.)

5. Selected studies did not specify that patients with dementia were suffering from other morbidities or not, and also did not explain that patients might be completely paralyzed or partially paralyzed or were quite stable if in the early stage of dementia.

Response: This is correct. Information on other morbidities was not available in the studies included in this systematic review. While these persons with dementia were living in the community (rather than institutional care), we cannot make assumptions about their functioning.

6. Studies did not specify the age of caregivers and gender. Age and gender also bring different responses.

Response: Age and gender of caregivers have now been added to the table in the “family caregivers” column. Please note that an error was found in the reporting of the total number of caregivers in the Grey-Vickery (1993) study. There are now 11 caregivers in this study, which necessitated an edit in the table and an edit in the description of the total number of participants on line 194.

7. In discussion, there is a need to describe findings from formal (family members) and informal careers separately.

Response: As in response to #4 above, this systematic review only describes family caregivers. This highlights the confusion that can result with the term “informal carers” when it can refer to family caregivers in some places while it can refer to other people who provide help in the support network (paid or unpaid). The term “informal carer” has not been used throughout the manuscript to lessen this confusion. A wording change in lines 355-356 has been provided to be clearer.

B. **Responses to points raised by each reviewer:**

Reviewer #1:

The only concern is the stereotyping with the use of "rural persons, rural caregivers of rural families" as found on lines 256, 284, 313, 358-360. These should be revised to read " caregivers of persons/families resident in rural communities."

Response: Thank you for this suggestion. The term was revised as suggested by the reviewer on lines 107-108, 267-268, 296-297, 324-326, 372-373.

Reviewer #2:

This paper needs to revise in every section. Need to write the paper according to the template of the journal. Please check the organization of the paper. The updated references should be added. A few of the research directions should be implemented.

Response: Thank you for these comments. The paper has been written according to the template of the journal and it has been checked for organization. Each section was reviewed for required revisions.

Reviewer #3:

1. In paper, a cohort of studies was selected as there is only one study published in 1993 which is mentioned in Table (1), while the remaining 35 studies are from 2000-2022. How was this cohort decided? If researchers intended to add research published in 2000-22 then they should exclude 1993.

Response: There were no study exclusions based on year of publication. The 1993 study was a dissertation that resulted from our searches, and it was highly relevant to the purpose. All other studies were published from 2000 and on, but there was no reason to exclude the 1993 study based on the eligibility criteria.

2. In paper, study rationale and implication are missing. Add some paragraphs about study implications and significance.

Response: Thank you for the opportunity to provide more information. Please see lines 96-112 for rationale, implications, and significance.

3. Researchers selected studies from Canada (n=15), the 187 United States (n=7), Australia (n=3), Scotland (n=3), Norway (n=1), Sweden (n=1), South Africa 188 (n=1), Kenya (n=1), Ghana (n=1), and Japan (n=1). As Japan is an Asian country then researches from other Asian countries like Pakistan, India, Sri Lanka, China, Iran, Malaysia, were not included? kindly write down the reasons behind selection of countries.

Response: No countries were excluded from this systematic review. There were no other countries represented in this list because there were no more studies which met the inclusion criteria.

4. Researchers just gave overall findings of researches, at least they should specify the category of caregiver, no doubt, in method section, they have described caregiver” was defined as an unpaid career who may be referred to as a care partner or informal career; But in Table 1, a separate column should be made for the extracted themes to elaborate that family caretaker might have different concerns than those informal careers.

Response: This systematic review is solely on the experience of family caregivers, also described as unpaid family caregivers or carers. A change was made to line 142 to be clearer. (We recognize the confusion that results when there is no one term for unpaid family caregivers and the term “informal” caregiver can mean a paid person outside the family. Thus, the article is only addressing family members and the reference to care partner or informal caregiver was removed for ease of understanding.)

5. Selected studies did not specify that patients with dementia were suffering from other morbidities or not, and also did not explain that patients might be completely paralyzed or partially paralyzed or were quite stable if in the early stage of dementia.

Response: This is correct. Information on other morbidities was not available in the studies included in this systematic review. While these persons with dementia were living in the community (rather than institutional care), we cannot make assumptions about their functioning.

6. Studies did not specify the age of caregivers and gender. Age and gender also bring different responses.

Response: Age and gender of caregivers have now been added to the table in the “family caregivers” column. Please note that an error was found in the reporting of the total number of caregivers in the Grey-Vickery (1993) study. There are now 11 caregivers in this study, which necessitated an edit in the table and an edit in the description of the total number of participants on line 194.

7. In discussion, there is a need to describe findings from formal (family members) and informal careers separately.

Response: As in response to #4 above, this systematic review only describes family caregivers. This highlights the confusion that can result with the term “informal carers” when it can refer to family caregivers in some places while it can refer to other people who provide help in the support network (paid or unpaid). The term “informal carer” has not been used throughout the manuscript to lessen this confusion. A wording change in lines 355-356 has been provided to be clearer.

Please let me know if you have any further questions about our submission.

Sincerely,

Heather J. Campbell-Enns, PhD

Associate Professor, Department of Psychology

Canadian Mennonite University, Winnipeg, MB

hcampbell-enns@cmu.ca
